# The prokineticin receptor agonist Bv8 decreases IL-10 and IL-4 production in mice splenocytes by activating prokineticin receptor-1

**DOI:** 10.1186/1471-2172-9-60

**Published:** 2008-10-28

**Authors:** Silvia Franchi, Elisa Giannini, Donatella Lattuada, Roberta Lattanzi, Hui Tian, Pietro Melchiorri, Lucia Negri, Alberto E Panerai, Paola Sacerdote

**Affiliations:** 1 Department of Pharmacology, University of Milan, Via Vanvitelli 32, 20129 Milano, Italy; 2 "V. Erspamer" Department of Human Physiology and Pharmacology, "La Sapienza" University, Piazza Aldo Moro, Rome, Italy; 3 Amgen, South San Francisco, California, USA

## Abstract

**Background:**

Bv8, prokineticin-1, or endocrine gland-vascular endothelial growth factor, and prokineticin-2 are recently isolated peptide agonists of two G protein-coupled receptors, prokineticin receptor-1 (PROKR 1) and PROKR 2, and have been described as affecting a number of myeloid cell functions. We evaluated the impact of Bv8 on lymphoid cells by investigating its ability to modulate T cell cytokine balance in mouse.

**Results:**

The production of T-helper1 cytokines (IL-2, IFN-γ and IL-1β), the T-helper 2 cytokine IL-4, and the anti-inflammatory cytokine IL-10 by mouse splenocytes was evaluated after polyclonal stimulation or immunisation with the keyhole limpet hemocyanin protein antigen by measuring cytokine levels. When added *in vitro *to Con-A-stimulated splenocytes, Bv8 significantly increased IL-1β and decreased IL-4 and IL-10; IL-2 and IFN-γ were not affected. Similar results were obtained when Bv8 was administered *in vivo*. In KLH-immunised mice, splenocytes restimulated *in vitro *with KLH and Bv8 produced significantly smaller amounts of IL-4 and IL-10. KLH-induced IL-10 and IL-4 production was also significantly blunted in animals administered Bv8 *in vivo *at the time of KLH immunisation or two weeks later. The Bv8-induced effects were lost in mice lacking the PROKR 1 gene, thus indicating that PROKR 1 is the receptor involved in the modulation of cytokines.

**Conclusion:**

These findings indicate that Bv8/prokineticin-1 is a novel modulator of lymphoid functions, and may be a suitable target for new immunopharmacological strategies.

## Background

The small 77-amino acid protein Bv8 belongs to a novel family of secreted proteins [1], and was given its name to indicate its origin (the frog *Bombina variegata*) and molecular weight (8 kD). Its orthologues have been conserved throughout evolution from invertebrates to humans: the mammalian orthologues are mouse Bv8 or prokineticin(PROK) 2 and PROK 1, and humans have Bv8-like proteins called prokineticin-1 (PROK 1) or endocrine gland-vascular endothelial growth factor (EG-VEGF), and human Bv8 or PROK 2 [2-4]. It has been shown that Bv8 and the mammal analogues have the same activity [5].

The mRNAs of the murine Bv8-like proteins PROK 1 and 2 have been detected in the brain, spinal cord, dorsal root ganglia, gastrointestinal tract, endocrine glands, spleen and circulating leukocytes of mice, rats and humans [4,6,7]. Two receptors for this family of secretory proteins (PROKR 1 and PROKR 2) have been identified in humans, rats and mice [8,9]; they belong to the G protein-coupled receptor family, share approximately 85% amino acid identity, and are distributed in brain and the peripheral organs, including the spleen and leukocytes [7,10,11].

The list of biological activities associated with Bv8/PROK peptides is rapidly growing. They seem to influence complex behaviours, such as feeding and drinking, and circadian rhythms, and they are involved in neuronal survival, angiogenesis, and the reproductive cycle [2,3,6,12-16]. It has also been clearly demonstrated that Bv8 has potent hyperalgesic activity [1,10,11,17].

It is now emerging that the immune system may be an important target for this family of peptides. LeCouter *et al*. [7] have shown that Bv8 and EG-VEGF are related to the regulation of hematopoiesis and hematopoietic cell mobilisation, and Dorsch *et al*. [18] that they are involved in monocyte differentiation and activation.

We have recently characterised the effect of Bv8 on murine macrophages, and shown that the protein can induce a pro-inflammatory macrophage phenotype [19]: it stimulates macrophage chemotaxis and increases the production of the pro-inflammatory cytokines IL-1β and IL-12, while decreasing that of IL-10. It is also well known that, in addition to being important regulatory cells in innate immunity, macrophages participate in the development of T-helper (Th) cells to Th1 or Th2 [20]. IL-12 is the critical factor driving the development of Th1 cells, which are associated with cell immune responses and tissue injury [20-23], whereas the Th2 cells responsible for humoral responses and allergies are stimulated by IL-4, which is produced by Th2 lymphocytes themselves [21,22,24]. IL-10 is a major anti-inflammatory cytokine that is produced by monocytes/macrophages, as well as by distinct subsets of T cells that include Th2 and several populations of the recently identified Treg [21,25,26]. Given the effect of Bv8 on macrophages, it can be expected that the cytokine milieu induced by Bv8 also has an impact on T cell-derived cytokines.

For these reasons, after confirming the presence of PROKRs on splenocyte-derived T lymphocytes, we analysed the effects of the *in vitro *and *in vivo *administration of Bv8 on splenocyte cytokine production in mice. Th1 cytokines (IL-1β, IFN-γ and IL-2), the Th2 cytokine IL-4, and IL-10 were evaluated after polyclonal mitogen activation, and in mice immunised with the protein antigen keyhole limpet hemocyanin (KLH). In order to identify the type of receptor involved, we also carried out some experiments using cells obtained from PROKR 1 knock-out (KO) mice.

The *in vitro *and *in vivo *activation of PROKR 1 by Bv8 significantly decreased IL-4 and IL-10 production, without directly affecting the Th1 cytokines, thus indicating that the prokineticin system may compromise anti-inflammatory immune responses.

## Methods

### Materials

Bv8 was isolated from skin secretions of the frog *Bombina variegata*, and was purified to 98% as assessed by means of HPLC [1].

### Animals

Balb/CJ mice (Charles River, Calco, Italy) weighing 20–25 g were housed at 22 ± 2°C in an environment with a light/dark cycle of 12 h, and allowed food and water *ad libitum*.

The characteristics of PROKR 1 KO mice have been previously described in detail [11].

Briefly, our PROKR 1 KO mice (Lexicon Genetics Inc., The Woodlands, TX) were generated by constructing a targeting vector in which exon 1 of the PROKR 1 gene was replaced with a neomycin resistance gene derived from the LacZ/Neo vector. Lex-1 embryonic stem cells were electroporated with the targeting vector before the cells expressing the targeted allele were selected for the generation of the chimerae, which were then bred with C57BL/6 mice. The progeny were genotyped by means of PCR, which permitted the amplification of the wild-type (WT) PROKR 1 gene (5'-GGTGACTATGACATGCCCCTGG-3', 5'-CTCTCGGAAAGGGAGAGGCAAGG-3') and the neomycin-resistant gene cassette, which was inserted to disrupt the PROKR 1 coding region (5'-CAGCGCATCGCCTTCTATC-3', 5'-CTCTCGGAAAGGGAGAGGCAAGG-3'). Genomic DNA was isolated from tail samples by means of proteinase K (Sigma) digestion and ethanol precipitation, and 200 ng DNA was amplified (HotStarTaq DNA Polymerase, Qiagen) using the following cycle parameters: 95°C for 3 min (1 cycle); 95°C for 1 min, 55°C for 1 min, 72°C for 1 min (30 cycles); and 72°C for 10 min (1 cycle). The amplified products were resolved on 2% agarose gel.

WT littermates were used as controls.

All of the animal procedures were performed in accordance with the Italian and European regulations governing the care and treatment of laboratory animals (Permit No. 94/2000A), and approved by the Institutional Review Board of Milan University's Department of Pharmacology.

### Collection of splenocytes, and the production of concanavalin-A(Con-A)-induced cytokines

The spleens of the animals were aseptically removed, and 20-gauge sterile needles were used to tease the cells through an incision made in the spleen cuticle [27,28]. The cells were adjusted in 24-well plates to 4 × 10^6 ^cells/ml of medium (RPMI supplemented with 10% FCS, 1% glutamine, 2% antibiotics, 0.1% 2-ME), and incubated at 37°C in 5% CO_2 _and 95% air in the presence or absence of 10 μg/ml Con-A for 24 h (in the case of IL-1β, IL-2 and IFN-γ) or 48 h (in the case of IL-10 and IL-4), which are the times of maximum cytokine release [27,28].

In the *in vitro *experiments, Bv8 at concentrations ranging from 10^-7 ^to 10^-13 ^M was added to the wells at the same time as Con-A. When the PROKR 1 KO and WT mice were used, the cells were isolated as described above, and Bv8 was added at a concentration of 10^-9 ^M.

In the *in vivo *experiments, Bv8 was administered subcutaneously (s.c.) in the flank at a dose of 250 pmoles/kg, and the spleens were obtained four hours later. The cells were then incubated with or without the mitogen. The 4-hour interval between Bv8 administration and spleen harvesting was chosen on the basis of previously published data concerning the pharmacodynamics and pharmacokinetics of Bv8, which show that it modulates a number of biological functions at this time [10,29].

### KLH immunisation

The mice were injected intraperitoneally (i.p.) with 100 μg of the protein antigen KLH (Calbiochem, La Jolla, California) in a volume of 0.2 ml of saline; 14 days after immunisation, they were decapitated and their spleens were aseptically removed. The cells were obtained as described above, and plated at a concentration of 7 × 10^6 ^in 24-well plates containing a final concentration of 80 μg/ml KLH in a total volume of 1 ml.

The presence of *in vitro *KLH restimulates the KLH-specific T-lymphocyte clones previously activated by *in vivo *KLH. This is a frequently used method for inducing cytokine production by specific T-lymphocyte clones [27].

In the *in vitro *experiments, Bv8 (10^-9 ^M) was added to the wells alone or together with KLH. The plates were incubated at 37°C in 5% CO_2 _and 95% air, and the supernatants were collected after 48 h (in the case of IL-2 and IFN-γ) or 72 h (in the case of IL-10 and IL-4), which are the times of maximum cytokine release [27]; they were then stored frozen at -80°C for cytokine analysis. The KLH concentration used *in vitro *(80 μg/ml) was chosen on the basis of previous experiments [27] showing that it induces sub-maximal and easily measurable cytokine production in such a way as to allow the detection of any stimulation or inhibition induced by Bv8 treatment.

In the *in vivo *experiments, Bv8 was administered s.c. in the flank at a dose of 250 pmoles/kg at the same time as KLH, or 14 days after KLH immunisation and four hours before spleen cell harvest. Bv8 was administered at these times in order to identify when it exerts its modulatory activity during the immunisation process: at the time of antigen recognition or at the time of final effector events. The 4-hour interval between Bv8 administration and spleen harvesting was chosen on the basis of previously published data concerning the pharmacodynamics and pharmacokinetics of Bv8, which show that it modulates a number of biological functions at this time [10,29]

The control animals were immunised with KLH and treated with saline instead of Bv8 at the same times. The cells were then stimulated *in vitro *with KLH.

The same protocols were used for the PROKR 1 KO and WT mice.

### Cytokine ELISA

The levels of IL-2, IL-4, IL-10 and IFN-γ were determined by means of a standardised ELISA protocol (Pharmingen, San Diego, CA). The anti-IL-2 and IL-4 (1 μg/ml) and anti-IL-10 and IFN-γ (2 μg/ml) capture monoclonal antibodies (mAbs) were absorbed on a polystyrene 96-well plate, and the cytokine in the sample was bound to the antibody-coated wells; after this, biotinylated anti-IL-10, IL-4 and IL-2 detecting mAbs (0.5 μg/ml) and the anti-IFN-γ mAb (1 μg/ml) were added to bind the cytokine captured by the first antibody. After washing, avidin-peroxidase (Sigma) was added to the wells in order to detect the biotinylated detecting antibody and, finally, the 2,2'azino-bis(3-ethylbenzthiazoline6-sulfonic acid) (ABTS, Sigma) substrate was added. A coloured product was formed in proportion to that measured at an optical density 405 nm. The standard curves ranged from 15 to 2000 pg/ml for IL-2, IL-4 and IL-10, and from 32 to 4000 pg/ml for IFN-γ. The IL-1β levels were measured by mean of an Endogen kit (Tema Ricerca, Bologna. Italy) in accordance with the manufacturer's instruction. The standard curve ranged from 15 to 1000 pg/ml.

### Measurement of PROKR 1 and PROKR 2 mRNA expression

In order to obtain purified spleen cell populations for the real-time RT-PCR experiments, after erythrocyte lysis, the splenocytes were appropriately stained with PE anti CD11b for the mieloyd line, PE-anti-CD3 antibody for T lymphocytes and FITC anti CD19 for B lymphocytes (Miltenyi Biotec, Bologna, Italy) and the CD11b^+^, CD3^+ ^CD19^+ ^populations were purified (purity ≥ 95%) using a BD FACSAria sorter (BD Biosciences, San Diego, CA) a method that has been previously described in detail [30,31]. In order to obtain sufficient cells for the RNA extractions, spleens from 3–6 mice were used for each staining.

Real-time quantitative PCR was performed as previously described [11]. Briefly, total RNA was prepared from sorted cells using the RNeasy micro Kit (Qiagen, Milan, Italy) and quantitated using the Ribo Green RNA Quantitation Kit (Invitrogen, San Giuliano Milanese, Italy) according to manufacturer's instructions. Real-time RT-PCR was performed using a iCycler apparatus (BIO-RAD, Segrate, Italy) and the Quantitect SYBR one-step RT-PCR kit (Qiagen, Milan, Italy), supplemented with 0.3 μM of each primer pair for PROKR 1 or PROKR 2 [11] and 60 ng of total RNA to give a final reaction volume of 25 μl. The cycling conditions were 30 min at 50°C; 15 min at 95°C; and then 50 cycles of 30 s at 95°C, 30 s at 52°C and 30 s at 72°C. The individual specific PCR products were confirmed by means of melting curve analysis and gel electrophoresis, and the results were normalised to 18S RNA expression levels within each sample [11]. The Δ*C*_T _value was determined by subtracting the aver age 18S *C*_T _value from the average PROKR 1, 2 and Bv8 *C*_T _in the same sample. The levels of both receptors in the different cell populations were expressed in relation to the most expressed value, and relatively quantified by means of the comparative method: the amount of target, normalised to an endogenous reference and relative to a calibrator (mean of the most expressed gene ΔC_T_).

### Statistical analysis

The results were expressed as mean values ± S.D, and analysed using one-way ANOVA followed by Bonferroni's *t*-test for multiple comparisons. A P value of <0.05 was considered statistically significant.

## Results

### Analysis of PROKR 1 and PROKR 2 expression in splenocytes

**Figure 1:**
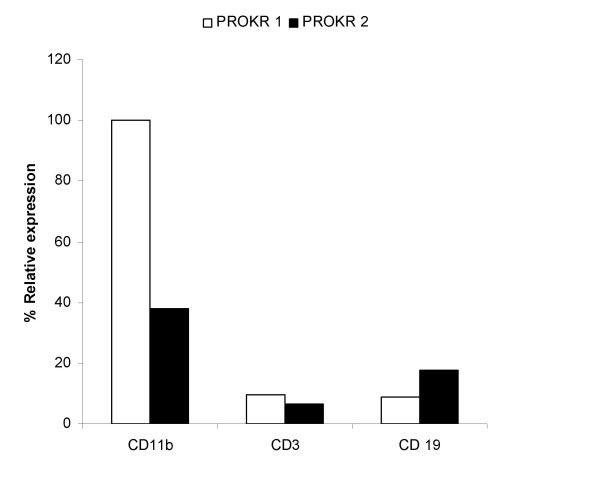
**PROK receptors**. PROKR 1 and PROKR 2 are expressed in murine spleen-derived cells. RNA was extracted from CD11b^+^, CD3^+ ^and CD19^+ ^splenocytes and relative expression of PROKRs was evaluated by real time-RT-PCR.

The expression of PROKR 1 and PROKR 2 in most murine immune cell types has been previously reported [18,19] however, we used real-time RT-PCR to confirm their presence in the splenocytes used in this study (Figure 1). We found specific transcripts for PROKR 1 and PROKR 2 in CD11b^+^, CD3^+ ^and CD19^+ ^splenocyte-extracted RNA. The highest PROKR expression was observed in CD11b+ cells. In these cells we observed a predominance of PROKR 1 transcript in comparison to PROKR 2. Lower and similar expression of both receptors was found in CD3^+ ^and in CD19^+ ^cells, with a slight predominance of PROKR 2 in B lymphocytes.

### Effects of Bv8 on Con-A-stimulated cytokine production *in vitro *and *in vivo*

**Figure 2:**
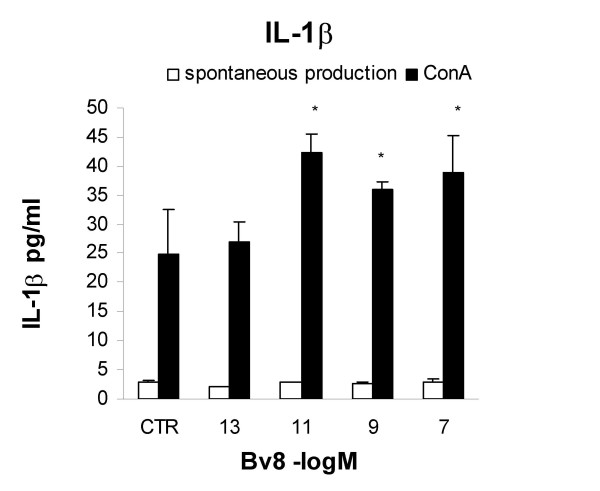
**IL-1 production**. Effect of the *in vitro *addition of Bv8 on IL-1β production by mouse splenocytes in the presence or absence of 10 μg/ml Con-A; IL-1β levels were measured 24 hours later. Results from one typical experiment involving eight mice/group. Mean values ± SD. *p < 0.05 *vs *CTR (Con-A stimulated cultures without Bv8).

Bv8 was added to the *in vitro *splenocyte cultures with or without Con-A in order to evaluate IL-1β, IL-2, IFN-γ, IL-10 and IL-4 production. As shown in Figure 2, the spontaneous production of IL-1β was very low and not affected by Bv8 which, however, significantly boosted IL-1 production in the splenocytes stimulated by Con-A. The increase was clear at Bv8 concentrations of 10^-11^, 10^-9 ^and 10^-7 ^M, but the lowest concentration of 10^-13 ^was not effective.

**Figure 3:**
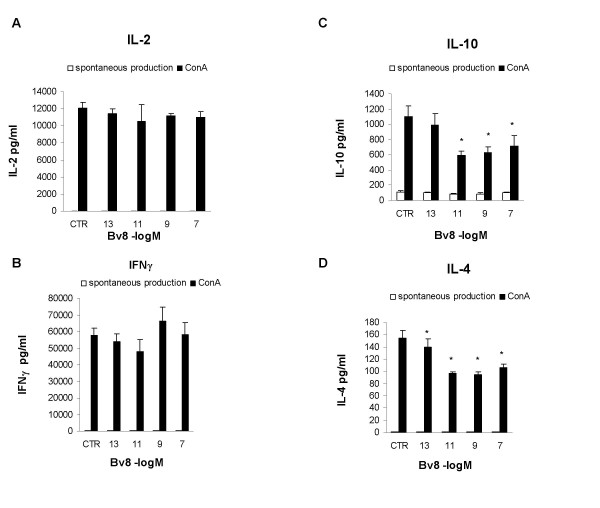
**In vitro Bv8**. Effect of the *in vitro *addition of Bv8 on IL-2 (panel A), IFN-γ (panel B), IL-10 (panel C) and IL-4 production (panel D) by mouse splenocytes cultured in the presence or absence of 10 μg/ml Con-A for 24 h (IL-2 and IFN-γ) or 48 h (IL-10 and IL-4). Results from one typical experiment involving eight mice/group. Mean values ± SD. *p < 0.05 *vs *CTR (Con-A stimulated cultures without Bv8).

Panels A and B in Figure 3 show the concentrations of the Th1 cytokines, IL-2 and IFN-γ, which were low when the cells were cultured without mitogen, but increased after the addition of Con-A; none of the tested Bv8 concentrations had any effect. However, as shown in panel C, the *in vitro *addition of Bv8 significantly reduced Con-A induced IL-10 production at concentrations of 10^-11^, 10^-9 ^and 10^-7 ^M.

Unstimulated cells did not produce any measurable IL-4 with or without Bv8 but, when stimulated by Con-A, they produced a substantial amount, which was significantly reduced by the addition of Bv8 at all of the tested concentrations (Fig. 3, panel D).

**Figure 4:**
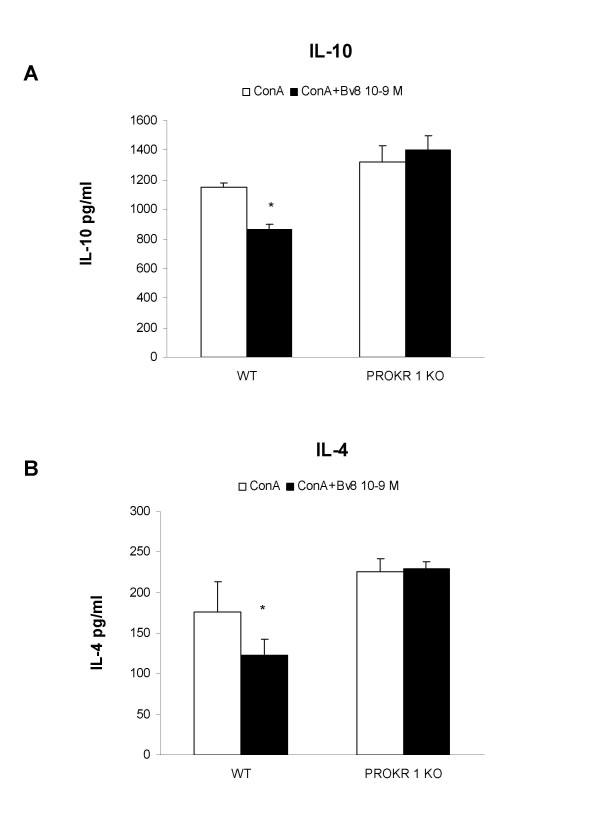
**PROKR 1 KO mice**. Effect of the *in vitro *addition of Bv8 (10^-9 ^M) on IL-10 (panel A) and IL-4 production (panel B) by mouse splenocytes from WT and PROKR 1 KO mice. The cells were stimulated *in vitro *with Con-A for 48 h. Results from experiments involving four mice/group. Mean values ± SD. *p < 0.05 *vs *Con-A only.

Figure 4 shows the effects of Bv8 on Con-A-stimulated cytokine production in PROKR 1 KO mice and WT controls. The production of cytokines by the cells obtained from the WT controls was slightly different than that measured in those obtained from the Balb/CJ mice (see Figure 3), but this is not surprising as they are different strains. Bv8 10^-9 ^M lowered IL-10 (panel A) and IL-4 concentrations (panel B) in the WT controls in the same way as in the Balb/C mice, but the fact that these effects were completely abolished in the PROKR 1 KO mice demonstrates their dependence on the receptor.

**Figure 5:**
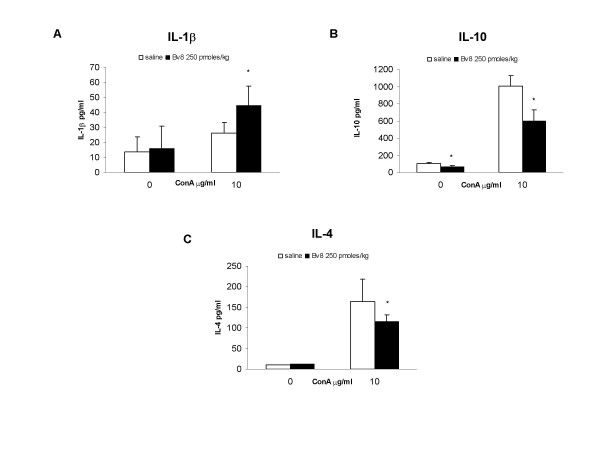
**In vivo Bv8**. Effect of the *in vivo *administration of Bv8 250 pmoles/kg on the splenocyte production of IL-1β, IL-10 and IL-4. The splenocytes were obtained 4 h after Bv8 treatment and stimulated *in vitro *in the presence or absence of 10 μg/ml Con-A for 24 h (IL-1) or 48 h (IL-10 and IL-4). Results from one typical experiment involving eight mice/group. Mean values ± SD. *p < 0.05 *vs *saline-treated animals.

In order to check the *in vivo *ability of Bv8 to modulate cytokine levels, it was injected s.c. at a dose of 250 pmoles/kg and, four hours later, splenocytes were obtained and cultured with or without Con-A. The results were similar to those observed when Bv8 was added *in vitro*. As shown in Figure 5 (panel A), the levels of Con-A-induced IL-1β were significantly increased by the administration of Bv8, whereas the production of IL-2 and IFN-γ seemed to remain unchanged (data not shown).

The levels of unstimulated and Con-A-induced IL-10 were significantly lower in the culture medium of the splenocytes obtained from the Bv8-treated mice than in that of the splenocytes obtained from the saline-treated animals (Fig. 5, panel B). Significantly lower amounts of IL-4 were also found in the Con-A-stimulated cultures of splenocytes harvested from the Bv8-treated animals (Fig. 5, panel C).

### Effects of Bv8 on KLH-stimulated Th1 and Th2 cytokine production *in vitro *and *in vivo*

**Figure 6:**
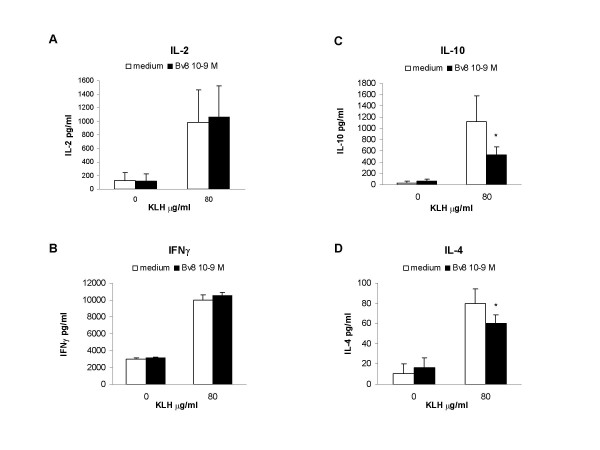
**KLH immunisation + in vitro Bv8**. Effect of *in vitro *addition of Bv8 (10^-9 ^M) on IL-2 (panel A), IFN-γ (panel B), IL-10 (panel C) and IL-4 production (panel D) by splenocytes of mice immunised with KLH. Two weeks after immunisation, the splenocytes were cultured in the presence or absence of 80 μg/ml KLH for 48 h (IL-2 and IFN-γ) or 72 h (IL-10 and IL-4). Results from one typical experiment involving eight mice/group. Mean values ± SD. *p < 0.05 *vs *KLH-stimulated cultures without Bv8.

Mouse splenocytes obtained two weeks after the animals were immunised with KLH were restimulated *in vitro *with KLH 80 μg/ml, which induced the substantial production of IL-2, IFN-γ, IL-10 and IL-4. The *in vitro *addition of Bv8 did not affect the production of IL-2 and IFN-γ(Fig. 6, panels A and B), but significantly reduced both IL-10 and IL-4 release (Fig. 6, panels C and D).

In a second series of experiments, Bv8 or saline was administered *in vivo *to mice at the time of KLH immunisation or two weeks later, four hours before the spleen cells were harvested for *in vitro *restimulation with KLH. Bv8 was administered at these times in order to identify when it exerts its modulatory activity during the immunisation process: at the time of antigen recognition or at the time of final effector events.

**Figure 7:**
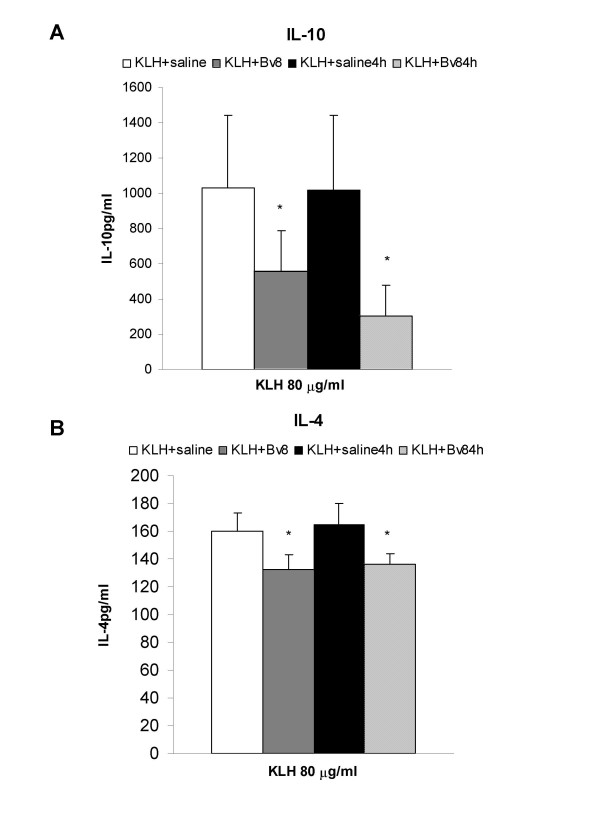
**KLH immunisation + in vivo Bv8**. Effect of the *in vivo *administration of Bv8 250 pmoles/kg on the production of IL-10 (panel A) and IL-4 (panel B) by splenocytes of mice immunised with KLH. Two weeks after immunisation, the splenocytes were cultured in the presence or absence of 80 μg/ml KLH for 72 h. Bv8 was administered at the time of immunisation (KLH + Bv8) or 4 h before splenocyte collection two weeks later (KLH +Bv4 h). Results from one typical experiment involving eight mice/group. Mean values ± SD. *p < 0.05 *vs *corresponding saline-treated animals.

The levels of IL-2 and IFN-γ produced by the KLH-stimulated splenocytes obtained from the mice treated with Bv8 were no different from those measured in the control animals (data not shown), but the levels of IL-10 were significantly lower regardless of whether Bv8 was administered at the time of KLH immunisation or four hours before *in vitro *KLH restimulation (Fig. 7, panel A). Similarly, IL-4 production also decreased after Bv8 administration at either time (Fig. 7, panel B).

**Figure 8:**
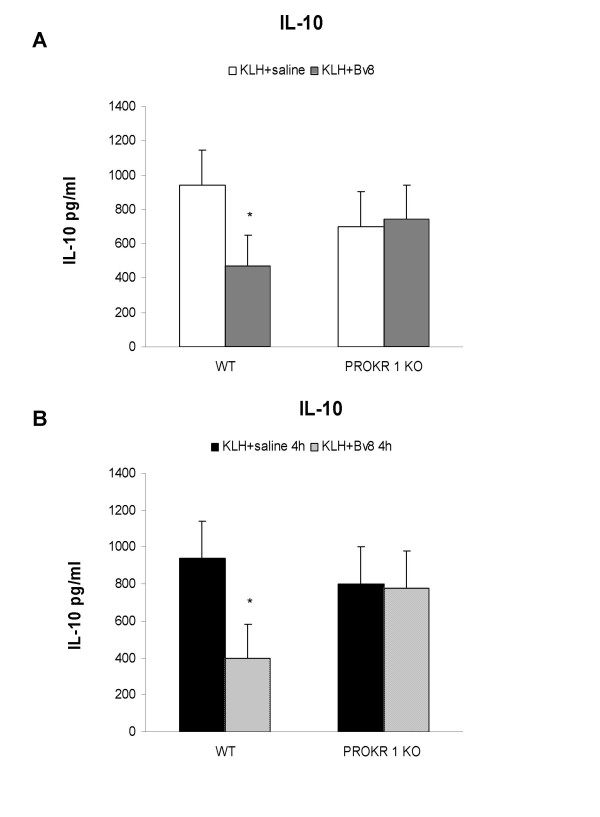
**KLH immunisation in PROKR 1 KO mice**. Effect of the *in vivo *administration of Bv8 250 pmoles/kg on the production of IL-10 by splenocytes of WT and PROKR 1 KO mice immunised with KLH. Two weeks after immunisation, the splenocytes were cultured in the presence or absence of 80 μg/ml for 72 h. Bv8 was administered at the time of immunisation (panel A) or 4 h before splenocyte collection two weeks later (panel B). Results from typical experiments involving four mice/group. Mean values ± SD. *p < 0.05 *vs *saline-treated animals of the same strain.

In order to assess the type of PROK receptor involved in the Bv8-induced decrease in IL-10 levels, PROKR 1 KO mice were immunised with KLH and treated *in vivo *with Bv8 at the same time (Fig. 8, panel A) or two weeks later, four hours before the collection of splenocytes (Fig. 8, panel B). As shown in Figure 8, and as observed in the BalbC mice, the *in vivo *administration of Bv8 at either time significantly decreased IL-10 production in the WT animals but not in the in the PROKR 1 KO mice.

## Discussion

Our results show that the newly discovered Bv8/prokineticin system plays a role in modulating T cell function by reducing Th2 cytokine levels and IL-10 production, thus indirectly switching the cells towards a Th1 and pro-inflammatory state. We used amphibian Bv8 as it is a potent agonist of PROKR 1 and PROKR 2, and is widely accepted as a means of studying the effect of PROKR activation in mammals [5,11].

We and others have previously demonstrated that the Bv8 system has a significant impact on the expression of monocyte and macrophage cytokines by suppressing IL-10 production and significantly increasing the production of pro-inflammatory cytokines and IL-12 [7,18,19]. Our present findings showing the modulation of T-lymphocyte cytokines indicates that the PROK system is involved in modulating both innate and acquired immunity.

The results obtained after *in vitro *stimulation with the polyclonal mitogen Con-A, which binds to T lymphocyte membrane glycoproteins (including TCR and CD3) but has no stimulatory effect on other immune cell types, indicate that Bv8 acts directly on the T lymphocyte population in the spleen. It also modulates Con-A-induced cytokines when administered *in vivo *four hours before Con-A *in vitro *stimulation, a situation in which its effect could be mediated by its activity on other cell types.

Our KLH experiments indicate that Bv8 affects a number of temporal steps in immune activation: the *in vivo *experiments showed that it significantly modulates IL-4 and IL-10 production regardless of whether it is given at the time of KLH immunisation or four hours before *in vitro *KLH restimulation. The effect of Bv8 is profound and long-lasting as a single administration is sufficient to shape the response measured 14 days later. These findings suggest that it probably acts on the early events involving naïve T cells that take place during antigen recognition, such as antigen presentation by dendritic cells.

However, the modulation observed four hours before *in vitro *stimulation indicates that Bv8 also acts on the activation and proliferation that occur when T cells encounter antigen *in vitro*. This effect is confirmed by our *in vitro *experiments showing that Bv8 also decreases IL-10 and IL-4 levels when added to splenocytes at the time of antigen restimulation. On the whole, it seems that the effect of Bv8 on the Th2 cytokines IL-4 and on IL-10 may not only be due to direct interactions with T lymphocytes, but may also be mediated by other cell types such as macrophages [19].

However, our preliminary cytofluorimetric analysis seems to indicate that IL-10 production is decreased mainly in CD3^+ ^lymphocytes [Franchi et al., unpublished results].

IL-4 is the hallmark cytokine of the Th2 phenotype, but IL-10 is secreted by Th2 cells and a number of the recently identified Treg populations that play a crucial role in limiting inflammation [25,26]. It can be hypothesized that Bv8 affects the induction of specific IL-10-producing T cells, but future detailed studies are needed to characterise the T lymphocytes targeted by Bv8 to reduce IL-10 production.

Two receptors of the family of secretory proteins (PROKR 1 and PROKR 2) have been identified in humans, rats and mice [8,9]. They belong to the G protein-coupled receptor family, share approximately 85% amino acid identity, and are distributed in brain and the peripheral organs, including the spleen, and leukocytes [7-11,18,19].

In particular, Dorsch *et al*. [18] have demonstrated the presence of both receptors on T and B lymphocytes, as well as on macrophages purified from murine spleen, and we have also shown [19] the high expression of PROKR 1 in murine peritoneal macrophages. We now confirm that specific transcripts for both receptors are present in the purified spleen-derived macrophages, T and B lymphocytes. Both PROKR 1 and 2 are more highly expressed in CD11b+ cells than in T and B lymphocytes; moreover in this cell type a predominance of PROKR 1 transcripts over PROKR 2 is found. This last observation is consistent with our previous results in peritoneal macrophages [19], although does not completely agree with other data showing a relative prevalence of PROKR 2 [18]. Consistently with the data reported by Dorsch et al., [18], equal PROKR 1 and PROKR 2 expression appears to be present in T lymphocytes, while PROKR 2 is slightly more abundant in B cells. A recent review [32] on the presence of PROKRs in the immune cells, has suggested that the distribution of the two receptors can differ between human and murine cells and that further analysis are needed. As PROKRs are present on all cell types present in the spleen, it is likely that Bv8 regulates cytokine production binding to multiple immune cells. However, the loss of effect of Bv8 observed in our PROKR 1 KO mice suggests that PROKR 1 is the main receptor involved in modulating immune responses, which is in line with our previous findings indicating that it is exclusively responsible for mediating the modulation of macrophage cytokine production [19]. Moreover, considering the high expression of this receptor on cells of the myeloid lineage, it can be hypothesized that the Bv8 immune effects are due to the activation of this population, rather than to a direct effect on T lymphocytes. This aspect will be evaluated in future work.

As immune cells produce PROK, possess functional receptors and respond to Bv8, prokineticins may play a role as autocrine/paracrine regulators of immune responses.

Bv8 is highly expressed in mouse and human immune cells, and has been reported in mouse and human monocytes, macrophages, granulocytes, spleen and bone marrow [7,18,19]. We also confirm that purified CD3 lymphocytes also express Bv8 mRNA (Franchi et al, unpublished data). Furthermore, prokineticins are significantly overexpressed in inflamed tissues and, in line with this, it has been found that Bv8, PROK and their receptors are all involved in inflammatory processes and mediate inflammatory pain [10,11]. It has very recently been shown that the implantation of tumour cells in mice upregulates Bv8 in serum, bone marrow and spleen myeloid cells, and that G-CSF is a positive major regulator of Bv8 expression [5]. It can be hypothesised that the Bv8 produced by myeloid cells under these different conditions acts as a cytokine on T cell functions.

A disrupted balance of pro- and anti-inflammatory cytokine production, and alterations in the induction of the different T cell populations, are involved in the development of various immune diseases [25,33,34], and so the effects of Bv8 on different types of T cell may play a broad pathophysiological role. Moreover, recent studies have begun to identify some peptide antagonists of PROKR 1 receptors [35,36] that may offer a novel approach to the development of drugs designed to modulate immune/inflammatory responses.

## Conclusion

Various activities have been associated with Bv8 signalling, and this family of proteins is emerging as an important new modulator of immune responses. We show that Bv8 plays a specialised role in modulating T cell cytokine production as it significantly reduces IL-4 and IL-10. Given the important role that both cytokines play in limiting inflammation, it can be suggested that the Bv8/PROK system plays a general pro-inflammatory role, which means that Bv8 or its receptors may represent new therapeutic targets.

## Authors' contributions

SF performed most of the experiments and participated in writing the manuscript

EG prepared the Bv8 for the study and reviewed the manuscript

DL performed the FACS experiments

RL performed some of the *in vivo *experiments and generated the PROKR 1 KO animals

HT generated the PROKR 1 KO animals

PM provided funding and reviewed the manuscript

LN originated the idea for the research and reviewed the manuscript

AEP critically analysed the data and reviewed the manuscript

PS designed the study, supervised the work, provided funding and participated in writing the manuscript

All of the authors have read and approved the final manuscript
